# Growth failure of unknown etiology

**DOI:** 10.20945/2359-4292-2026-0113

**Published:** 2026-10-05

**Authors:** Robert Rapaport

**Affiliations:** 1 Icahn School of Medicine at Mount Sinai, Pediatric Endocrinology, New York, New York, United States

Dear Editor,

I read with interest and pleasure your communication about “Healthy Short Stature” (^1^).

For several years I have been writing about my displeasure with the term “idiopathic short stature”, in large part, not so much because it’s idiopathic, but because it focuses on stature rather than growth (^2^). For that reason, I have been much in favor of the terms that you referenced (^3^,^4^) as idiopathic growth failure to emphasize that it’s the process of growth that’s impaired that needs to be looked at rather than just stature. Correcting stature alone leaves one open to the criticism of treatment for cosmetic reasons alone.

Professors Savage, Wit and I (^5^) emphasized that a thorough evaluation needs to be done before one resolved that it is “idiopathic short stature”. I agree with the comments in your publication that once a genetic diagnosis, monogenic or polygenetic, is made that individual no longer has “ISS” but a certain condition that also affects growth and stature. I have proposed that the term “growth failure of unknown ideology GFUE”, be used for patients currently labeled as ISS. Certainly “idiopathic growth failure IGF” would be ideal, but the initials IGF are already taken by another entity.

With your permission, I have some reservations about your proposal of “Healthy Short Stature”. I am concerned about the term “healthy” for a child whose height is 2 1/2 to 3 standard deviation below the mean who is likely not growing sufficiently, but in whom no cause for his or her stature and growth is yet demonstrated. He or she may may have a condition the etiology of which is not yet known. The term healthy to me at least indicates that there is absolutely no problem and that the patient never needs to seek medical attention again contrary to what you indicate in your presentation that “this nomenclature preserves and even reinforces the need for care for clinical assessment and judicious application of genetic testing”. Certainly, I think that in the United States if indeed you are healthy, the implication is that you no longer need to seek any medical attention, except perhaps for general health maintenance by a primary care provider. Growth and stature are no longer issues to be concerned about. Healthy short stature would also imply that that’s a condition to which one should aspire. Does the “healthy short stature” become less healthy if the person grows well? Therefore, I am respectfully suggesting that the application of the term, healthy short stature at least in the families that I have been dealing with would result in no longer attending physicians or anyone else in the healthcare system for this condition. I think that would be detrimental. Labeling the child “healthy” and the resultant lack of any further investigations may lead to never identifying etiologies we currently still do not know.

Alternative for otherwise healthy children with growth issues have been previously, and by some still, referred to as “normal growth variants”, specifically constitutional growth delay and familial short stature (^6^). Neither of these conditions was excluded from the initial ISS consensus.

## Data Availability

datasets related to this article will be available upon request to the corresponding author.
